# Education, training, and professional issues of radiographers in six European countries: a comparative review

**DOI:** 10.3402/jecme.v5.31092

**Published:** 2016-04-05

**Authors:** Andreas G. Prentakis, Antonis P. Stefanoyiannis, Kostas Georgiadis, Louise Coleman, Shane J. Foley, Daniela Herlig, Photis Kollas, Anna Kowalik, Jolanta Tomczak, Sofia N. Chatziioannou

**Affiliations:** ^a^ Nuclear Medicine Department, University General Hospital “ATTIKON”, Athens, Greece; ^b^ The Society and College of Radiographers, London, United Kingdom; ^c^ School of Medicine & Medical Science, University College, Dublin, Ireland; ^d^ Swiss Association of Radiological Technologists, Lucerne, Switzerland; ^e^ Radiology Department, Mediterranean Hospital of Cyprus, Limassol, Cyprus; ^f^ Medical Physics Department, Greater Poland Cancer Centre, Poznan, Poland; ^g^ Department of General and Vascular Surgery, Poznan University of Medical Sciences, Poznan, Poland

**Keywords:** radiographer, education, training, practice, continuing professional development

## Abstract

Radiographers constitute an important part of a multidisciplinary radiation-based imaging and therapy chain. However, is there a common framework for assuring high education, training, and subsequent practice of profession among European countries? A study was conducted, based on a questionnaire that consisted of three parts, concerning education and training (Part A), national registry (Part B), and professional issues (Part C). Analysis of the collected data suggested that a common policy is generally followed in the countries investigated; however, differences were not negligible. A common framework of educational programmes among European countries could form the basis for overall standardisation at national and international level.

## Introduction

The use of ionising radiation in everyday clinical practice has flourished for more than 3 decades.^1^ Advances in technology offer several benefits to the patients, having as a prerequisite a sufficient education and training level of the involved personnel.^2^ Most radiation-based diagnostic examinations and therapeutic practices require multidisciplinary collaboration. Radiographers belong to a team of professionals who work together to ensure that the corresponding medical procedures are carried out effectively and safely. A radiographer has the flexibility to work in a variety of fields, including radiology, nuclear medicine, and radiotherapy departments. A standardised level of education and training is expected to enhance effectiveness, in accordance with European directives.^3^ Furthermore, professional mobility is a fundamental goal of current European policies. Such policies aim at the establishment of a professional qualifications framework and a European Qualification Framework for Lifelong Learning.^4^–(6)

This study aims at the structured classification of information concerning the education and training of radiographers in different, geographically spread European countries, as well as the subsequent clinical practice of the profession. Data analysis is expected to reach conclusions regarding the minimum educational and training requirements and the professional role of radiographers in each country.^7^

## Methods

Based on the general structure of similar studies regarding medical physicists and nuclear medicine physicians, a questionnaire has been electronically sent to 21 registered professional societies.^8^–(11) The questionnaire was written in English and consisted of three parts with 15 questions in total. In addition, it was forwarded to radiographers who were national delegates, to ensure responses were as accurate and as reliable as possible.

Part A (entitled “Education and training”) examined the required type and level of degree (University or not), the duration of a certified study programme, and the temporal contribution of practical training. Part B (entitled “National Registry”) pertained to the existence of a national registry of radiographers, the role of continuing professional development (CPD), and the possibility of acquiring a postgraduate degree within national boundaries.^12^ Finally, in Part C (entitled “Professional issues”), each country representative was asked about the necessity of holding a professional license, the prospect of specialisation of radiographers, and the mandatory physical presence of other health care professionals during the accomplishment of certain aspects of their duties.

The educational and training systems vary among countries investigated, but a common categorisation to post-secondary and tertiary or university level was used. Educational systems were also chronologically classified as undergraduate and postgraduate.^13^ Simple descriptive statistics were used to summarise the results.

## Results

Six countries responded (Cyprus, Greece, Ireland, Poland, Switzerland, and the United Kingdom), which amounts to a 29% response rate.

### Part A: Education and training

Part A is analysed in Table [Table T0001] and 2 [Table T0002]. In all countries, a nationally approved education and training programme exists with a mandatory study duration of 2–4 years. Apart from Cyprus, every country offers a Bachelor of Science (BSc) degree (3–4 years duration). However, an alternative to acquiring a non-tertiary degree is offered in Cyprus, Greece, Poland, and the United Kingdom, by attending a post-secondary school for 1–3 years. Practical clinical training duration is 20–50% of the whole study duration in all countries. Furthermore, Greece offers the opportunity to work as a radiographer by completing a 2-year programme without clinical practice (post-secondary education level), after being certified for sufficient knowledge of basic radiation standards. A similar mechanism exists in the United Kingdom, where a diploma of assistant practitioner is awarded after 1–2 years of studies and subsequent certification by the College of Radiographers (CoR).

**Table 1 T0001:** Issues regarding the educational programme of radiographers

Part A: Education and training	Required degree to practise the profession and corresponding educational level	Study duration in years	Theoretical and clinical time distribution

Cyprus	BSc or non-university level	3	80 and 20%
Greece	BSc or post-secondary level	4 (public school) or 2 (private school)	55 and 45% (no clinical training for post-secondary level)
Ireland	BSc (level 8)	4	70 and 30%
Poland	BSc or non-university level (2 years of study at medical school)	2 (studying at medical school, after national exams) or 3 (BSc)	50 and 50%
Switzerland	Tertiary level or BSc	3	50 and 50%
The United Kingdom	BSc (Hons) or PG Diploma/MSc. Assistant practitioner – Foundation degree	1–2 (as an assistant practitioner) or 3–4 for BSc (Hons)	50 and 50%

**Table 2 T0002:** Issues regarding the training programme of radiographers

Part A: Education and training	Are training centres nationally accredited? Which is the official body responsible for the accreditation?

Cyprus	Faculties accredited by the Ministry of Education
Greece	Faculties accredited by the Ministry of Education
Ireland	University programmes accredited by the CORU, the state regulator for health and social care professionals. The universities are both externally and internally accredited as part of the National University of Ireland
Poland	Accreditation by Ministries of Education and Health
Switzerland	Faculties are accredited by the National Authority of Vocational Education and Technology
The United Kingdom	Professional body is the College of Radiographers (CoR). Registering body is the Health and Care Professions Council (HCPC). All pre-registration courses are approved by the CoR and HCPC

All educational faculties of the countries investigated are accredited by national bodies that were either established for this purpose (as in Ireland and the United Kingdom) or constitute an official organisation in general (as in Greece). These organisations are also responsible for the curriculum. However, participating hospitals are generally not accredited.

### Part B: National registry

The answers concerning Part B are presented in Table 3. In Cyprus, Ireland, Switzerland, and the United Kingdom, national organisations that keep a registry of radiographers have been established. In United Kingdom, this organisation (Health & Care Professions Council – HCPC) is responsible for the biannual audit and the renewal of the registered radiographers, incorporating CPD accreditation. In Ireland, a similar system of registry renewal and CPD is present. Except for Cyprus and Switzerland, the other countries investigated offer a wide choice of postgraduate programmes and the opportunity to acquire the degree of Master of Science (MSc) and Doctor of Philosophy (PhD).

**Table 3:** National registry and Continuing Professional Development (CPD) mechanisms of radiographers

Part B: National registry	Is there a compulsory registry of radiographers? How is someone registered? Is there a renewal mechanism of the registry? Does it incorporate CPD procedures?

Cyprus	There is a registry and compulsory registration with annual renewal No CPD incorporation
Greece	No national registry present
Ireland	There is a registry and compulsory registration by CORU, the state regulator for Health and Social Care Professions with annual renewal and CPD incorporation audited biannually
Poland	No national registry present
Switzerland	The Swiss Red Cross mandated by the National Authority of Vocational Education and Technology (Bundesamt für Berufsbildung und Technology) registers every diploma/bachelor degree issued in Switzerland. It is voluntary with no renewal mechanism and CPD incorporation
The United Kingdom	Registration of radiographers by HCPC (compulsory). Registration of assistant practitioners by CoR (voluntary). A total of 2.5% of radiographers have their CPD audited every 2 years (HCPC). The CoR provides CPD accreditation (renewable every 2 years). Assistant, advanced, and consultant practitioners are re-accredited biannually

CPD=continuing professional development.

### Part C: Professional issues

In Table 4, Part C is analysed. In all six countries, an official body offering a professional license exists. Radiographers in Ireland and the United Kingdom can work in all fields and modalities. Working in radiotherapy in Ireland requires a different educational pathway, while in the United Kingdom, further training is essential. Ultrasound imaging is performed by radiographers in Ireland but not in the other countries investigated. Finally, in all countries except Cyprus and the United Kingdom, the close supervision of a medical doctor or a medical physicist is mandatory for the radiographer to carry out certain tasks. These tasks include mainly preparation and administration of I.V. agents, such as contrast media and radiopharmaceuticals.

**Table 4:** Professional issues of radiographers

Part C: Professional issues	License for radiographers (legal requirement, accreditation path, official body involved)	Ability to work on any modality	Legal requirement for carrying out duties under supervision

Cyprus	License accredited by the official body responsible for radiographers	Yes (except ultrasound)	No
Greece	License accredited by the Ministry of Health	Yes (except ultrasound)	Yes
Ireland	License is certified by CORU, the State Regulator for Health and Social Care Professions	Working in radiotherapy is possible after successful completion of a different educational pathway. Radiographers can work on any other modality	No
Poland	License is accredited by the Ministries of Education and Health to professionals holding BSc and/or MSc degrees	Yes (except ultrasound)	Yes
Switzerland	License is accredited by National Authority of Vocational Education and Technology	Yes (except ultrasound)	Yes
The United Kingdom	Licenses are certified by CoR and HCPC	In any department (further education and training may be required)	No

## Discussion

This study revealed that there is a similar study duration among the countries investigated. However, a BSc-level degree is not offered in all countries. The establishment of the radiographers’ degree to university level is expected to improve the quality of the services provided, even if extension of study duration is required. This upgrade could aim essentially to deal with current weaknesses of each country's educational system.^14^ For example, the undergraduate cycle is proposed to last not less than 3 years.^5^

Each educational programme incorporates part of practical clinical training. Students’ training in a clinical environment is highly encouraged.^15^ The mean duration of practice in certified hospitals of these six countries exceeds 40% of the whole educational programme with a median value of 50%. An official threshold of practice duration should be defined following consensus of the corresponding national professional societies. Hospitals should be accredited for this role by official bodies, in order to offer high standards of training. Trainees should preferably practise in all modalities in these hospitals, under the supervision of certified personnel. Successful completion of clinical training should be objectively quantified by means of trainees’ active involvement in a minimum number of various radiation-based procedures currently applied in agreement with European guidelines concerning medical specialties.^16^

Most countries investigated have already established a license for many specialties, including that of radiographers. An accredited license to work as a radiographer is expected to further protect public health, since acquiring such a certification will assure possession of certain qualifications and skills.

Among the countries investigated, only the United Kingdom and Ireland have applied a fully organised registry of radiographers, with an incorporated CPD scheme. Such an approach should set an example for the other countries, so that professionals are able to remain up-to-date in the field.^17^ The CPD system offers the motivation to acquire new knowledge, to develop new skills of patient management, and assure provision of high quality of services.^18^

In all countries, a radiographer is able to work in most imaging and therapeutic modalities. The freedom of choice is considered beneficial to the employee, because it provides the opportunity to work in many fields, therefore, expanding job skills and improving overall professional performance.^19^ Moreover, radiographers’ ability to work in different modalities facilitates the department's workflow, in cases of special needs.

In general, radiographers are not allowed to work in ultrasound departments (with the exception of Ireland and the United Kingdom), mainly because ultrasound diagnosis is considered to be the preserve of the medical staff. In addition, drug administration is considered to be a medical intervention; hence, qualified personnel should be available during this procedure if needed.

This study should be interpreted in light of certain limitations. First, the number of countries that responded was relatively low (almost 30% response rate). This may be attributed to the strict time frame (1 month) that was set to the countries’ representatives, in order to assure that the recorded status for all countries corresponded to the same time period. In similar future studies, the response rate may be remedied by setting a less strict time frame for data collection and/or forwarding the questionnaire to more than one representative per country. In addition, the study could be further expanded by forwarding a similar questionnaire to countries of other continents, properly adjusted to account for possible terminology differences. Second, the exact content of education and training programmes was not examined, along with the possible requirements for completion of the specialty, apart from time-related thresholds. In future studies, data could be collected concerning a more detailed view of educational programmes. As an example, active participation to a predefined number of diagnostic or therapeutic procedures and/or carrying out research could be considered as an essential ingredient for completion of training.^16^

## Conclusions

A questionnaire consisting of 15 questions was prepared and utilised, in order to organise the structured classification of data concerning education and training of radiographers, as well as the subsequent practice of the profession, in six European countries. The study demonstrated that a common policy is generally followed in the countries investigated despite the presence of a few differences. Suggestions are made to include standardisation of the educational level, the accreditation of clinical training, with active involvement of radiographers in a minimum number of procedures and the adoption of CPD systems and national registries renewal mechanisms. Expansion of this survey to a greater number of countries is expected to confirm the similarities and differences noted, providing the basis for the overall standardisation of educational programmes at national and international level.

## Conflict of interest and funding

The authors declare that they have no conflicts of interest concerning this article.
